# Scattering integral equation formulation for intravascular inclusion biosensing

**DOI:** 10.1038/s41598-024-64633-0

**Published:** 2024-07-01

**Authors:** Constantinos Valagiannopoulos, Daniele Tosi

**Affiliations:** 1 School of Electrical and Computer Engineering, National Technical University of Athens, 15772 Athens, Greece; 2 School of Engineering and Digital Sciences, Nazarbayev University, Astana, 010000 Kazakhstan

**Keywords:** Nanobiotechnology, Sensors and probes, Optical physics

## Abstract

A dielectric waveguide, inserted into blood vessels, supports its basic mode that is being scattered by a near-field intravascular inclusion. A rigorous integral equation formulation is performed and the electromagnetic response from that inhomogeneity is semi-analytically evaluated. The detectability of the formation, based on spatial distribution of the recorded signal, is estimated by considering various inclusion sizes, locations and textural contrasts. The proposed technique, with its variants and generalizations, provides a generic versatile toolbox to efficiently model biosensor layouts involved in healthcare monitoring and disease screening.

## Introduction

Molecular diagnostics have been substantially assisted by nanomaterials, namely, arrays of plasmonic nanoparticles^1^, in the quest for practical, robust, and highly sensitive detection agents that can address the deficiencies of conventional technologies^2^. In particular, surface plasmon resonance spectroscopy makes a ubiquitous method for probing the binding of biomolecules through the changes in refractive index occurring on thin metal films^3^ or via intensity concentrations around edges^4^. One may also refer to silicon nanowire label-free nanosensors^5^ achieving a significant overlap between the probing field and the active biological substances by supporting guided modes along their axes^6^. Moreover, graphene, in planar^7^ or rolled^8^ form, can make a tunable plasmonic biosensor for chemically-specific detection of protein monolayers, especially when subjected to nitrogen doping^9,10^. Finally, rapid imaging of cancer cells can be performed with help from quantum dots^11^ and silicon nanobiotechnology^12^, via exploitation of narrowband resonances in aptamer receptors^13^ or through surface-enhanced Raman scattering^14^.

Intravascular biosensing makes a separate category of biological detection that requires increased accuracy and rules out destructive testing; therefore, phosphorescence lifetime imaging microscopy^15^ and pressure transducers^16^ are employed instead. In addition, stents are used to measure blood glucose^17^, intravascular robots can warn about vascular damage^18^ while devices implanted through the jugular vein enable permanent, wireless pressure monitoring^19^. Dynamic estimation for optical absorption becomes also feasible by using photoacoustic measurements^20^ and precision-microfabricated fiber-optic probes are used for temperature sensing^21^. Alternatively, one can employ laser speckle contrast imaging in the visualization of implanted micro-robots in microvascular networks; indeed, by combining imaging data and computational simulations, successful estimations of fluid flow shear stresses within multiscale vasculature of varying complexity, are reached^22^. Furthermore, an intravascular fluorescence catheter detects cysteine protease activity in vessels of the size of human coronary arteries in real time^23^. Importantly, optical fibers^24^ are also utilized for similar purposes of protein detection^25^, imaging of intravenous biofilms^26^ or refractive index sensing^27^ since they admit easy light injection and remote operation^28^.

Integral equations constitute a powerful tool towards understanding the photonic interactions of objects with certain backgrounds like optical fibers. Techniques to treat electromagnetic scattering by dielectric cylinders of arbitrary cross section^29^ or three-dimensional inclusions with noncanonical shape^30^, have been developed and assisted by rigorous integral equation formulations. They also provide a versatile platform for analytical modeling of the radiation by buried conductors into layered media^31^ and the backscattering from randomly rough dielectric surfaces^32,33^. A technique for efficient solution of method of moments matrix equations employing characteristic basis function has been proposed^34^, while the scattering integral equation has been inverted to estimate the permittivity of a sample under specific environments^35,36^. Interestingly, numerical filtering is applied to the solution of integral equations encountered in indirect sensing measurements^37^ while similar models are calibrated for synthetic aperture radar data, under both electromagnetic polarizations^38^.

In this work, we use a rigorous integral equation formulation^39^ to model the electromagnetic interactions between a planar silicon fiber and an external object, embedded in a background with small permittivity contrast. The incoming light is concentrated in the middle of the slab, as dictated by its basic supported mode; however, its evanescent components are scattered by the inclusion and create detectable signal perturbations, proportional to the textural difference. The spatial profiles of the scattering response at several longitudinal observation planes may reveal the presence of the near-field object while correlations between the waveform peaks and the scatterer permittivity, size or location can be made. The system modeled in our work resembles inline detection of biomolecules with dimensions comparable or higher than the oscillating wavelength. These might be from viral particles such as poxviruses with size up to some hundreds of nanometers^40^ to cancer cells developed in the lungs^41^ (smaller than $5~{\upmu {\rm m}}$) or breast^13^ (bigger than $10~{\upmu {\rm m}}$). The adopted integral-equation-based approach is able to capture most of the features of a real-world experimental configuration and, at the same time, sophisticated enough to support analytical solutions. Unlike other extremely simplistic techniques, we advocate that the proposed method offers a unique pathway towards semi-analytical modeling for biosensing setups with a broad application spectrum from general healthcare monitoring and screening for disease to clinical analysis and in vivo operations to human organs.

**Figure 1:**
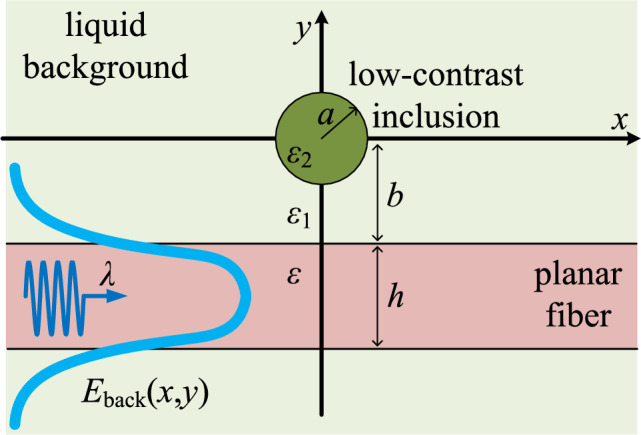
The layout of the considered setup. A planar fiber is located into liquid environment (blood) and guides a background electromagnetic field $E_{\rm back}(x,y)$ of wavelength $\lambda$. The leaked signal is scattered by a near-field cylindrical inclusion with low textural contrast.

## Results

### Input and output parameters

We consider the layout depicted in Fig. 1, where the used Cartesian coordinate system (*x*, *y*, *z*) is also defined; the associated polar coordinate system $(r,\varphi ,z)$ can be used instead. A planar slab of relative permittivity $\varepsilon$ and thickness *h* exists into an environment of relative permittivity $\varepsilon _1<\varepsilon$. The fiber guides its basic mode corresponding to free-space wavelength $\lambda =2\pi /k_0$ and creates a background electric field $E_{\rm back}(x,y)$ with vector parallel to *z* axis. This field is scattered by a cylindrical inclusion of radius *a* positioned at distance $b>a$ from the upper boundary of the slab. The gap size separating the inclusion from the dielectric waveguide is denoted as $g\equiv (b-a)$. The relative permittivity of the material filling the inhomogeneity is denoted by $\varepsilon _2$ and does not differ substantially from $\varepsilon _1$. In our formulation, the structure, the texture and the excitation is not dependent on *z*; therefore, we have a two-dimensional problem with the electric field *E*(*x*, *y*) possessing a single (*z*) component. All the permittivities $\{\varepsilon ,\varepsilon _1,\varepsilon _2\}$ are taken positive, but small imaginary parts indicating infinitesimal losses can be added for numerical reasons.

The total electric field, everywhere into the regarded space, comprises the background field $E_{\rm back}(x,y)$ and an extra term $E_{\rm scat}(x,y)$ produced by the inclusion, namely, $E(x,y)=E_{\rm back}(x,y)+E_{\rm scat}(x,y)$. The scattering component is given by the following integral^39,42^:

1 $$\begin{aligned} E_{\rm scat}(x,y) = k_0^2(\varepsilon _2-\varepsilon _1)\int _0^{2\pi }\int _0^a E(R,F)G(x,y,R,F)R{\text{d}}R {\text{d}}F, \end{aligned}$$

where *G*(*x*, *y*, *R*, *F*) is the scalar Green’s function of the considered layout for an observation point (*x*, *y*) expressed in the Cartesian coordinate system and for a source $(r,\varphi )$, expressed in the equivalent polar coordinate system, which is located into the region $y>-b$ along the axis $(r,\varphi )=(R,F)$. A similar formulation would be available if the scatterer was perfectly electrically conducting and supported surface currents around it^43–45^. The process of evaluating $E_{\rm scat}(x,y)$, based on the assumption $\varepsilon _2\cong \varepsilon _1$, is thoroughly described in the “Methods” section, where also the explicit form of the basic mode of the fiber $E_{\rm back}(x,y)$ is presented.

The operational wavelength is dictated by the used laser that feeds our fiber and, thus, it is kept fixed^46^ throughout our analysis at $\lambda =1.55~{\upmu {\rm m}}$ while the waveguide thickness can be well-approximated by $h=5\lambda$. As far as our silica-based^47^ dielectric film is concerned, we assume a permittivity that is equal to: $\varepsilon \cong 1.454^2$ while the liquid background either it is water, blood or cytoplasm^48^ can be taken with fixed permittivity: $\varepsilon _1\cong 1.331^2$. The texture of the inclusion can vary within the range $1.34^2<\varepsilon _2<1.42^2$ to cover various types of cells such as muscle, fat or mitochondrial^49,50^. The scatterer is considered with substantial size ($3\lambda<a<5\lambda$) and close to the fiber ($0.05\lambda<g<0.30\lambda$), to better demonstrate the proposed concept.

**Figure 2:**
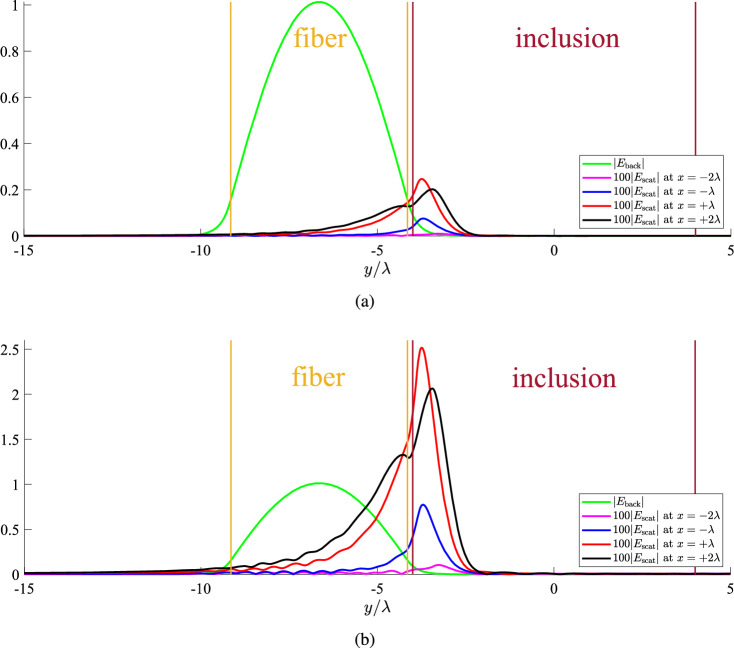
The background and 100-fold scattered field as functions of vertical distance $y/\lambda$ at several cross sections $x/\lambda$ for an inclusion with: (**a**) very small textural contrast $\varepsilon _2=1.34^2$, (**b**) moderate textural contrast $\varepsilon _2=1.42^2$. Common plot parameters: $a=4\lambda$, $g\equiv b-a=0.15\lambda$. The orange lines denote the boundaries of the fiber and the brown vertical lines the boundaries of the inclusion.

### Background vs scattering field profiles

In Fig. 2a, we represent the electric field profile along the transverse axis $y/\lambda$ at various longitudinal positions $x/\lambda$, for an obstacle with very low textural contrast $\varepsilon _2=1.34^2$. As indicated in the “Methods” section, the magnitude of the background field is not dependent on *x* since $E_{\rm back}\sim e^{-{\text{i}}\beta x}$ with $\beta \cong 1.45k_0$. In particular, the function $|E_{\rm back}(x,y)|$ is an even function of *y* around the center of the fiber $y=-b-h/2$, exhibits a unitary maximum exactly at this position, while vanishes exponentially outside of the waveguide. The orange vertical lines denote the boundaries of the Cartesian fiber while the brown ones show the spatial limits of the inhomogeneity at $x=0$. We also represent the 100-fold magnitude of the scattered electric field and realize that, for all the regarded constant-*x* planes, the maximum of $|E_{\rm scat}(x,y)|$ is exhibited for $|y|<a$, namely within the vertical limits of the inclusion. Importantly, the scattering effect is stronger along the forward ($x>0$) compared to the backward ($x<0$) direction while the perturbation dies more rapidly for $y>-a$ in contrast to what is happening for $y<-a$, where the background intensity is higher. Note that the scattering for $x=-2\lambda$ is negligible, while it boosts substantially for $x=\lambda$. It is important to stress that, despite the fact that $|\varepsilon _2/\varepsilon _1|\rightarrow 1$, the detection is feasible. Indeed, even though the peak of the scattering response barely reaches the 0.2% of the maximal input signal, it emerges at a position *y* that the background signal $|E_{\rm back}(x,y)|$ gets very weak too.

Biosensing is performed with even better terms in Fig. 2b, where the scatterer is characterized by a higher textural contrast, namely, $\varepsilon _2=1.42^2$. We note that the scaling is different and the total signal can be perturbed substantially in the presence of the inhomogeneity. Indeed, the background intensity is identical to that of Fig. 2a but the scattering responses are found much greater, regardless of the selection of *x* plane. Given the fact that the excitation field $|E_{\rm back}|$ is evanescent outside of the fiber ($|y-b-h/2|>h/2$), the scattered components appear more pronounced since they are compared with it. Once again, the influence of the inclusion on the recorded signal is more powerful for $x>0$ compared to $x<0$. Such a feature that can be attributed to the optically large size of the inclusion ($a=4\lambda$) which creates shading at the rear direction^51^, when illuminated with a wave $E_{\rm back}\sim e^{-{\text{i}}\beta x}$.

**Figure 3:**
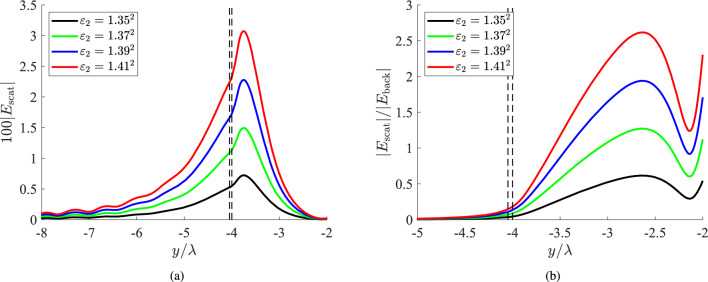
(**a**) The 100-fold scattered field and (**b**) the relative scattered field ratio $|E_{\rm scat}|/|E_{\rm back}|$, as functions of the vertical optical distance $y/\lambda$ for several permittivities of the inclusion, which is located very close to the fiber ($g\equiv b-a=0.05\lambda$). The black dashed lines denote the upper boundary of the slab and the lower boundary of the scatterer, namely, the distance between fiber and inclusion. Plot parameters: $x=\lambda$, $a=4\lambda$.

### Inclusions at different distances

In Fig. 3a, we represent the quantity $100|E_{\rm scat}|$ as a function of the electrical position $y/\lambda$ for several refractive indexes $\sqrt{\varepsilon _2}$ if the cylindrical obstacle is located in very close proximity to the fiber ($g\equiv b-a=0.05\lambda$). One directly notices stronger scattering compared to the ones examined in Fig. 2, surpassing even the 3% of the maximum (unitary) background intensity. As far as the variation is concerned, it exhibits peaks internally to the scatterer which, obviously, are higher the denser the inclusion gets (the larger the difference $|\varepsilon _2-\varepsilon _1|$ becomes). Therefore, we reach the anticipated conclusion that biosensing is easier for near-field obstacles.

In Fig. 3b, we consider the same cases as in Fig. 3a but we show the ratio $|E_{\rm scat}|/|E_{\rm back}|$ of the scattered field over the respective background field at the same position *x*. In this way, one can understand how much the scattering response will perturb the measured signal to reveal the presence and the features of the formation. Obviously, into the dielectric film the background field is dominant and, thus, the represented ratio vanishes. However, $|E_{\rm scat}|/|E_{\rm back}|$ increases exponentially outside the fiber to reach values larger than unity into the scatterer, before dropping again to give a local minimum. It is clearly noticed that, for sufficiently large textural contrast $|\varepsilon _2-\varepsilon _1|$, the scattered part can be up to three times higher than the excitation signal. One may wonder why the range along the horizontal axis $y/\lambda$ of Fig. 3b is different from that of Fig. 3a, even though they both refer to identical scenarios. The reason is related to the represented quantities: in Fig. 3b we show the ratio $|E_{\rm scat}|/|E_{\rm back}|$ which takes nonzero values outside of the fiber; on the contrary, the scattered electric field itself $|E_{\rm scat}|$ possesses non-negligible magnitudes, even when $y<-b$.

**Figure 4:**
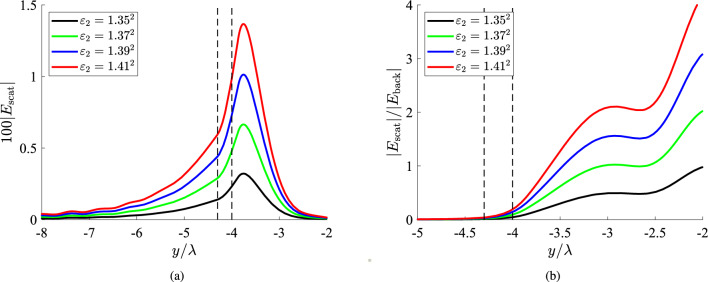
(**a**) The 100-fold scattered field and (**b**) the relative scattered field ratio $|E_{\rm scat}|/|E_{\rm back}|$, as functions of the vertical optical distance $y/\lambda$ for several permittivities of the inclusion, which is located at moderate distance from the fiber ($g\equiv b-a=0.3\lambda$). The black dashed lines denote the upper boundary of the slab and the lower boundary of the scatterer, namely, the distance between fiber and inclusion. Plot parameters: $x=\lambda$, $a=4\lambda$.

In Fig. 4a, we assume a more distant cylindrical inhomogeneity ($g=0.3\lambda$) and depict the spatial distribution of the quantity $|E_{\rm scat}(\lambda ,y)|$ for several $\varepsilon _2$. The average response from the scatterer is weaker compared to that of Fig. 3a since its source, namely, the object, is located farther. The peaks again appear into the formation but close to the dielectric waveguide, which justifies the placement of the biosensors in the spatial vicinity of the silicon. In Fig. 4b, we represent the respective ratios $|E_{\rm scat}|/|E_{\rm back}|$ as functions of $y/\lambda$ and we realize that they can obtain values close to 4, namely, larger that those of Fig. 3b. One may wonder how is it possible a lower $|E_{\rm scat}|$ to lead to more substantial ratio $|E_{\rm scat}|/|E_{\rm back}|$ given that $|E_{\rm back}|$ remains the same; it is explained by the fact that higher magnitudes for the ratio emerge at more distant *y* positions, where $E_{\rm back}$ gets almost vanished. For this reason, a very significant $|E_{\rm scat}|/|E_{\rm back}|$, as these emerging just beyond the local minima, may not necessary lead to successful detection; indeed, both $E_{\rm scat}, E_{\rm back}$ can be so tiny that get “drown” into the noise. At the same time, the scattering field $|E_{\rm scat}|$ should be strong enough to be recorded; that is why both variations of Fig. 4a, b are necessary to assess our ability for efficient biosensing. Based on the results presented in Fig. 4, the regarded setup can be utilized towards detection specificity intrinsic in the optical system, rather than via cell filtering such as in a lab-on-chip or by using bioreceptors^52^. For example, the refractive index difference between healthy tissue and ulcerated adenocarcinoma^53^ can give a substantial change in measured field intensity.

**Figure 5:**
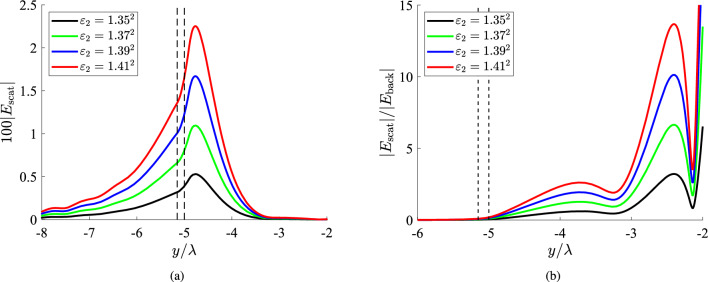
(**a**) The 100-fold scattered field and (**b**) the relative scattered field ratio $|E_{\rm scat}|/|E_{\rm back}|$, as functions of the vertical optical distance $y/\lambda$ for several permittivities of the inclusion, which is optically large ($a=5\lambda$). The black dashed lines denote the upper boundary of the slab and the lower boundary of the scatterer, namely, the distance between fiber and inclusion. Plot parameters: $x=\lambda$, $g\equiv b-a=0.15\lambda$.

### Inclusions of different sizes

In Fig. 5a, we once again represent the variation of $|E_{\rm scat}|$ along the *y* axis for a horizontal position $x=\lambda$ , which is selected based on the findings of Fig. 2 and an object of bigger size ($a=5\lambda$), compared to Figs. 3 and 4. The response is similar to those in Figs. 2, 3a and 4a, where the strength is proportional to the permittivity difference $|\varepsilon _2-\varepsilon _1|$ and vanishes within a couple of wavelengths $\lambda$ away from the bottom surface of the scatterer. In Fig. 5b, we sketch the quantity $|E_{\rm scat}|/|E_{\rm back}|$ for the same scenario of Fig. 5a, as we did in Figs. 3b and 4b. We realize that the recorded quantity can take extremely high values surpassing the limit of 15. However, such excessive ratios emerge at those positions *y* where $|E_{\rm scat}|$ is already negligible; therefore, the characteristics of the inclusions are not easily unveiled by the corresponding measurements.

**Figure 6:**
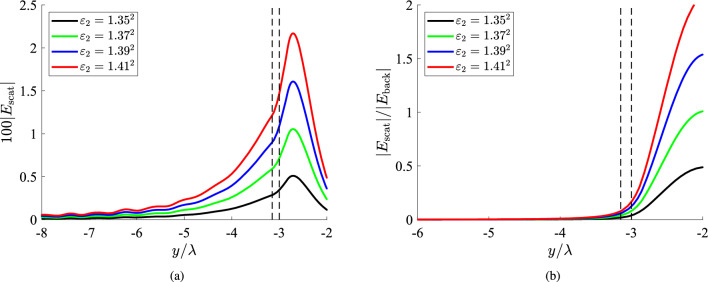
(**a**) The 100-fold scattered field and (**b**) the relative scattered field ratio $|E_{\rm scat}|/|E_{\rm back}|$, as functions of the vertical optical distance $y/\lambda$ for several permittivities of the inclusion, which is optically moderate in size ($a=3\lambda$). The black dashed lines denote the upper boundary of the slab and the lower boundary of the scatterer, namely, the distance between fiber and inclusion. Plot parameters: $x=\lambda$, $g\equiv b-a=0.15\lambda$.

In Fig. 6, we consider a smaller scatterer ($a=3\lambda$) and in Fig. 6a, we show the spatial distribution of $|E_{\rm scat}|$ with respect to $y/\lambda$. The obtained curves are very similar to those of Fig. 5a, meaning that what counts more is the distance *g* of the formation from the fiber and, of course, its permittivity $\varepsilon _2$, unlike radius *a*. Indeed, the maximal values of the data measurements for each $\varepsilon _2$ are almost identical to them in Fig. 5a. Such a feature demonstrates that increasing the size of the scatterer 2*a* beyond a threshold, does not render the object more easily detectable since the extra volume is surrounded by hugely evanescent fields, far away from the film. On the other hand, it is needless to say that the inhomogeneity should have a non-negligible volume to get sensed; once $a\ll \lambda$, the scattering response $|E_{\rm scat}|$ vanishes too. In Fig. 6b, the ratio $|E_{\rm scat}|/|E_{\rm back}|$ is sketched as a function of *y*, for the case of Fig. 6a. Once again, the quantity vanishes into the fiber as in Figs. 3b, 4b and 5b. Similarly, the ratio boosts sharply when the observation point exits the dielectric film and one directly notices that the depicted curves bear close resemblance to the respective ones of Fig. 5b but shifted by about $2\lambda$, equaling the difference in the radius *a* of the inclusion.

### Far-field response simulation

In order to demonstrate the sensing of the inclusion with an assortment of textural and structural characteristics, we numerically evaluate the scattered electric field in the far region (for observation points with $k_1 r \gg 1$), according to the process described in detail in the “Methods” section. The respective quantity is denoted by $|e_{\rm scat}(\varphi )|$; it simulates the far field response of the scatterer by dropping the radial (*r*) dependence and keeping only the azimuthal ($\varphi$) one.

**Figure 7:**
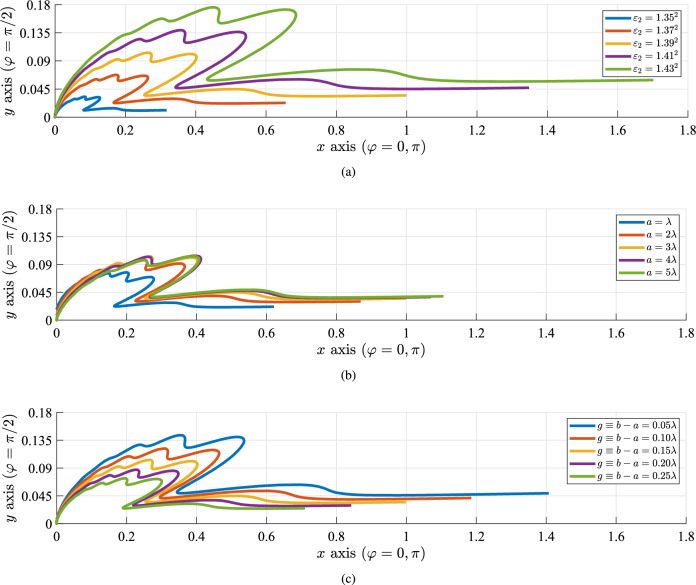
Normalized polar plots of the scattered far field $|e_{\rm scat}(\varphi )|$: (**a**) for various permittivities $\varepsilon _2$ of the inclusion ($a=3\lambda$, $g=0.15\lambda$), (**b**) for various optical sizes $a/\lambda$ of the inclusion ($\varepsilon _2=1.39^2$, $g=0.15\lambda$), (**c**) for various distances between the fiber and the inclusion ($a=3\lambda$, $\varepsilon _2=1.39^2$).

In Fig. 7a, we represent in polar plot a normalized version of $|e_{\rm scat}(\varphi )|$, divided by the same quantity, for several permittivities $\varepsilon _2$ of the inclusion. It is noteworthy that the response is much stronger along the forward scattering direction $\varphi =0$, while the backscattering ($\varphi =\pi$) is negligible. In addition, several radiation lobes are formed within the sector $0<\varphi <\pi /2$, which render the detection easier along specific rays by a receiver located at distant points. Obviously, the larger the textural contrast becomes, the more visible the object gets, based on far-field measurements. Such a conclusion is compatible with the reported results of Figs. 3, 4, 5 and 6. Moreover, in Fig. 7b, we show the same quantity of Fig. 7a in polar plot, but for various optical sizes $a/\lambda$ of the object this time. It is noted that the recorded signal is lower when the inclusion diameter is minimum and, naturally, the response gets amplified for more sizable objects. The scattering is particularly enhanced with $a/\lambda$ along $\varphi =0$ direction; that makes the sensing of the cylinder more direct, once probes are placed far from it but in the vicinity of the fiber. In Fig. 7c, we repeat the calculations of Fig. 7b, c by considering several distances $g/\lambda$ of the inclusions from the dielectric waveguide. The coupling is stronger for smaller gaps and, thus, the far-field scattering is increased once the inclusion is placed closer. Indeed, since the background mode is evanescent into the liquid background, the amount of power left to interact with the inclusion and develop a measurable response is decreasing exponentially with $g/\lambda$.

### Overview

An intravascular inclusion is considered in the spatial vicinity of a detecting fiber and its scattered field is semi-analytically evaluated with help from an integral equation formulation. The approach is rigorous and employs a singular and a smooth component for the Green’s functions, combined with Born approximation, based on the low textural contrast between the object and its environment. In particular, we assume that the field into the object is not affected substantially by itself and, accordingly, the obtained integrals are straightforwardly evaluated. The dielectric waveguide is excited by its basic mode and, thus, the near-field inclusion interacts only with the evanescent part of the background illumination. The scattered electric field is computed along planes vertical to the fiber for a variety of inclusion permittivities, sizes or distances from the dielectric film and is compared with the incident intensity. The object obviously becomes more easily detectable, the higher its permittivity difference with the background gets and the closer to the film is placed. However, as long as the scatterer is not tiny compared to the wavelength, its diameter does not play a crucial role since the field across the vast portion of it, vanishes.

The proposed technique can be expanded to study the detection of inclusions with multilayered or hollow fibers^54,55^; in such a scenario, the supported modes will admit higher radiation leakage and, accordingly, larger energy exchange with the objects in close proximity to the waveguide. In addition, a three-dimensional version of the same configuration comprising cylindrical fibers and spherical inclusions may be also solved semi-analytically by applying the entire-domain Galerkin technique, based on Mie-type spherical wave expansion of the field in the sphere and the use of dyadic Green’s function of the waveguide^56,57^. Importantly, the sensing of the bioparticles can be greatly assisted by adding nonlinearities to the utilized equipment; under proper selection of structural parameters one may achieve multistability in various geometries^58,59^; in this way, ultra-sharp changes in the response become possible and may reveal the presence and specific characteristics of the detectable objects.

## Methods

### Background field

With reference to Fig. 1, the background field is an even function with respect to $y=-b-h/2$ with a cosinusoidal profile into the fiber ($|y+b+h/2|<h/2$), written as: $E_{\rm back}=e^{-{\text{i}}\beta x}\cos \left( \sqrt{k^2-\beta ^2}\left( y+b+\frac{h}{2}\right) \right)$ while in the upper region ($y>-b$) is given by:

2 $$\begin{aligned} E_{\rm back}=e^{-{\text{i}}\beta x} \cos \left( \frac{h}{2}\sqrt{k^2-\beta ^2}\right) e^{-(y+b)\sqrt{\beta ^2-k_1^2}}. \end{aligned}$$

The symbols $k=k_0\sqrt{\varepsilon }$, $k_1=k_0\sqrt{\varepsilon _1}$, $k_0=2\pi /\lambda$ are used for the wavenumbers into the fiber, the background and vacuum respectively. The propagation constant $\beta$ is found by solving the equation imposing waveguidance^60^:

3 $$\begin{aligned} \tan \left( \frac{k_0h}{2}\sqrt{\varepsilon -\left( \frac{\beta }{k_0}\right) ^2}\right) = \frac{\sqrt{\left( \frac{\beta }{k_0}\right) ^2-\varepsilon _1}}{\sqrt{\varepsilon -\left( \frac{\beta }{k_0}\right) ^2}}, \end{aligned}$$

within the interval $\sqrt{\varepsilon _1}<\beta /k_0<\sqrt{\varepsilon }$. To achieve a single maximum of the background field in the middle of the fiber ($y=-b-h/2$), we select the largest root $\beta$ being as close as possible to the high end of the aforementioned range ($k_0\sqrt{\varepsilon }$).

### Green’s functions

The scalar Green’s function *G*(*x*, *y*, *R*, *F*) of the layout depicted in Fig. 1 for a source located into the region $y>-b$ along the axis $(r,\varphi )=(R,F)$ possesses different expressions in proportion to the position of the observation point: top ($y>-b$), middle ($-h-b<y<-b$) and bottom ($y<-b-h$). In the first case ($y>-b$), since both the source and the observation points belong in the same area, the Green’s function comprises two components, one singular and one smooth: $G=G_{\rm singular}+G_{\rm smooth}$. The singular term takes the form: $G_{\rm singular}(r,\varphi ,R,F)=-\frac{{\text{i}}}{4}\sum _{m=-\infty }^{+\infty }J_m(k_1\min (r,R))H_m(k_1\max (r,R))e^{{\text{i}}m (\varphi -F)}$, where $J_m$ is the Bessel function of order *m* and $H_m$ is the Hankel function of order *m* and second kind.

As far as the smooth term is concerned, it is written in a spectral integral form^36,43^:

4 $$\begin{aligned} G_{\rm smooth}(x,y,R,F)=\int _{-\infty }^{+\infty }C(\gamma ) e^{-{\text{i}}\gamma x-u_1(\gamma )y}e^{-{\text{i}}\gamma R\cos F-u_1(\gamma )R \sin F}{\text{d}}\gamma , \end{aligned}$$

where $u_1(\gamma )=\sqrt{\gamma ^2-k_1^2}$ is the radiation function evaluated with a positive real part. The explicit form of $C(\gamma )$ is not shown for brevity. Similar expressions like Eq. (4) are available for the Green’s functions when the observation point (*x*, *y*) lies in the middle or in the bottom region.

### Born approximation

The scattering integral^35,39^ gives the scattering field in terms of the integral of the Green’s function *G*(*x*, *y*, *R*, *F*) times the unknown electric field *E*(*x*, *y*) at the cross section of the cylindrical inclusion, multiplied by the textural contrast between the scatterer ($\varepsilon _2$) and the background ($\varepsilon _1$). In other words, $E_{\rm scat}=k_0^2(\varepsilon _2-\varepsilon _1)\int _{(S)} E G{\text{d}}S$, where (*S*), as indicated in Eq. (1), is the cross section of the cylinder. Given the fact that $\varepsilon _2\cong \varepsilon _1$, one can perform the so-called Born approximation by assuming that the electric field does not change significantly in the presence of the cylinder. Therefore, if $E(x,y)\cong E_{\rm back}(x,y)$, one obtains the following approximate expression for the scattered electric field:

5 $$\begin{aligned} E_{\rm scat}(x,y)\cong k_0^2(\varepsilon _2-\varepsilon _1)\int _0^{2\pi }\int _0^a E_{\rm back}(R,F)G(x,y,R,F)R{\text{d}}R {\text{d}}F. \end{aligned}$$

In order to evaluate the scattering response, we can directly substitute Eq. (2) in Eq. (5). For observation points into the upper region ($y>-b$), the integral of the singular term will be determined separately from that of the smooth term. On the contrary, if $y<-b$, only smooth components comprise the Green’s functions and will be integrated. Accordingly, the way of computing the expression (5) when $y>-b$ for $G=G_{\rm smooth}$ can be followed when the observation point lies elsewhere ($y<-b$). Therefore, in the following, we will only demonstrate the evaluation for the former case ($y>-b$).

### Singular scattering integral evaluation

If one considers the singular component of the Green’s function in Eq. (5), a series (with respect to *m*) of double integrals (with respect to *F* and *R*) are obtained. The azimuthal integration is analytically executed with help from the identity^36^:

6 $$\begin{aligned} \int _0^{2\pi } e^{-{\text{i}}\beta R \cos F-u_1(\beta ) R \sin F-{\text{i}}m F}{\text{d}}F=2\pi {\text{i}}^{-m}\left( \frac{\beta +u_1(\beta )}{k_1}\right) ^{-m}J_m(k_1 R), \end{aligned}$$

where $u_1(\gamma )=\sqrt{\gamma ^2-k_1^2}$. To this end, the radial integrations can be carried out with use of the formula:

7 $$\begin{aligned} \int _{\chi }^{\psi } J_m(k_1 R) Z_m(k_1 R) R {\text{d}}R= W_m^Z(\psi )-W_m^Z(\chi ), \end{aligned}$$

where:

8 $$\begin{aligned} W_m^Z(R)=\frac{R^2}{4}\left[ 2J_m(k_1 R) Z_m(k_1 R)-J_{m-1}(k_1 R)Z_{m+1}(k_1 R)-J_{m+1}(k_1 R)Z_{m-1}(k_1 R) \right] , \end{aligned}$$

while $Z=J,H$ can be the Bessel or Hankel function of order *m*. It is stressed that the radial integration is done within the interval $0<R<a$; therefore, for observation points with $r<a$, one should split the integral in $0<R<r$ and $r<R<a$ and then proceed with Eqs. (7), (8); indeed, the functions $\min (r,R), \max (r,R)$ are evaluated differently for each case. On the contrary, when $r>a$, we always have $r>R$ and thus $\min (r,R)=R$ and $\max (r,R)=r$.

In this way, the quantity $k_0^2(\varepsilon _2-\varepsilon _1)\int _{(S)} E_{\rm back}G_{\rm singular}{\text{d}}S$ is found as series with respect to *m* with general terms $D_m(r) e^{{\text{i}}m \varphi }$ possessing different explicit forms in proportion to the relative position of the observation point inside ($r<a$) or outside ($r>a$) the scatterer. Obviously, convergence checks for the series $\sum _{m=-\infty }^{+\infty }D_m(r)e^{{\text{i}}m \varphi }$ are performed every time we do the respective evaluations.

### Smooth scattering integral evaluation

As far as the smooth component of the Green’s function is concerned, appeared in Eq. (5), we have a triple integral with respect to the radial (*R*) and the azimuthal (*F*) at the cross section of the scatterer and the spectral variable ($\gamma$) as well. In this case, we make the radial integration first with use of the trivial integral:

9 $$\begin{aligned} \int _0^a e^{R K(\gamma ,F)} R {\text{d}}R=\frac{1+e^{a K(\gamma ,F)}(a K(\gamma ,F)-1)}{K^2(\gamma ,F)}, \end{aligned}$$

where $K(\gamma ,F)={\text{i}}(\gamma -\beta )\cos F-(u_1(\gamma )+u_1(\beta ))\sin F$. Therefore, the quantity $k_0^2(\varepsilon _2-\varepsilon _1)\int _{(S)} E_{\rm back}G_{\rm smooth}{\text{d}}S$ is found in terms of a double integral of a function as follows: $\int _{-\infty }^{+\infty } e^{-{\text{i}}\gamma x-u_1(\gamma )y}\int _0^{2\pi } f(\gamma ,F) {\text{d}}F {\text{d}}\gamma$. The azimuthal integration around a closed interval $0<F<2\pi$ is performed numerically with use of a large number of points that captures the angular variation of $f(\gamma ,F)$. The process is assisted by the fact that the integrand exhibits no singularity with respect to *F*.

When it comes to the spectral integration with respect to $\gamma$, it is again executed numerically with a sufficiently detailed representation of the integrands. It is noticed that extra convergence checks are necessary since the integration interval is infinite; however, the most important problem is that the integrand blows up at specific points of $\gamma$. Such a singularity issue is handled with the introduction of small losses to the employed media, an assumption that inherits a tiny imaginary part to the propagation constant $\beta$ satisfying Eq. (3). In this way, the poles of the integrand functions become slightly complex and the integral is evaluated without numerical snags. The same procedure can be followed for the computation of the scattering field $k_0^2(\varepsilon _2-\varepsilon _1)\int _{(S)} E_{\rm back}G{\text{d}}S$ for observation points into ($-h-b<y<-b$) or below ($y<-b-h$) the slab.

### Scattered far field

If the observation point is located externally to the inclusion, the scattered field owed to the singular component of the Green’s function, is written in the form: $\sum _{m=-\infty }^{+\infty }D'_m H_m(k_1 r)e^{{\text{i}}m \varphi }$. If the analytical expansion of Hankel functions for large arguments is taken into account^61^:

10 $$\begin{aligned} H_m(k_1 r)\cong \sqrt{\frac{2{\text{i}}}{\pi k_1 r}} {\text{i}}^m e^{-{\text{i}}k_1 r}, k_1r\gg 1, \end{aligned}$$

then the respective far field for distant observation points ($k_1 r\gg 1$), is approximated by:

11 $$\begin{aligned} k_0^2(\varepsilon _2-\varepsilon _1)\int _{(S)} E_{\rm back}G_{\rm singular}{\text{d}}S \cong \sqrt{\frac{2{\text{i}}}{\pi k_1 r}} e^{-{\text{i}}k_1 r} \sum _{m=-\infty }^{+\infty }D'_m {\text{i}}^m e^{{\text{i}}m \varphi }. \end{aligned}$$

As far as the scattering response produced due to the smooth component of the Green’s function is concerned, we will utilize the method of stationary phase^62^ for the following integral:

12 $$\begin{aligned} \int _{-\infty }^{+\infty } e^{-{\text{i}}\gamma x-u_1(\gamma )y}A(\gamma ) {\text{d}}\gamma \cong \int _{-k_1}^{k_1} e^{-{\text{i}}r\left( \gamma \cos \varphi +\sqrt{k_1^2-\gamma ^2}\sin \varphi \right) }A(\gamma ) {\text{d}}\gamma \cong \sqrt{\frac{2{\text{i}}}{\pi k_1 r}} e^{-{\text{i}}k_1 r} \frac{k_1 \pi }{\sqrt{2}} A(k_1\cos \varphi ), k_1r\gg 1, \end{aligned}$$

where $A(\gamma )$ is an arbitrary function of $\gamma$. Therefore, the corresponding field $\int _{-\infty }^{+\infty } e^{-{\text{i}}\gamma x-u_1(\gamma )y}\int _0^{2\pi } f(\gamma ,F) {\text{d}}F {\text{d}}\gamma$, is approximated by:

13 $$\begin{aligned} k_0^2(\varepsilon _2-\varepsilon _1)\int _{(S)} E_{\rm back}G_{\rm smooth}{\text{d}}S \cong \sqrt{\frac{2{\text{i}}}{\pi k_1 r}} e^{-{\text{i}}k_1 r} \frac{k_1 \pi }{\sqrt{2}} \int _0^{2\pi } f(k_1\cos \varphi ,F) {\text{d}}F, \end{aligned}$$

where the contribution from the spectral direction $\gamma =k_1\cos \varphi$ is the dominant one. By adding together the two terms Eqs. (11) and (13) and keeping only the azimuthal dependence, we have an expression for the axial field in the far region ($k_1 r\gg 1$):

14 $$\begin{aligned} e_{\rm scat}(\varphi ) \sim \sum _{m=-\infty }^{+\infty }D'_m {\text{i}}^m e^{{\text{i}}m \varphi } + \frac{k_1 \pi }{\sqrt{2}}\int _0^{2\pi } f(k_1\cos \varphi ,F) {\text{d}}F. \end{aligned}$$

## Data Availability

The datasets used and/or analysed during the current study available from the corresponding author on reasonable request.
